# Estimation of the Parameters of Burr Type III Distribution Based on Dual Generalized Order Statistics

**DOI:** 10.1155/2014/512039

**Published:** 2014-10-19

**Authors:** Chansoo Kim, Woosuk Kim

**Affiliations:** ^1^Department of Applied Mathematics, Kongju National University, Gongju 314-701, Republic of Korea; ^2^Department of Statistics and Actuarial Science, University of Iowa, Iowa City, IA 52242-1409, USA

## Abstract

The estimation of the parameters of Burr type III distribution based on dual generalized order statistics is considered by using the maximum likelihood (ML) approach as well as the Bayesian approach. The exact expression of the expected Fisher information matrix of the parameters in the distribution is obtained. Also, an approximation based on Lindley is used to obtain the Bayes estimator. To compare the maximum likelihood estimator and the Bayes estimator of the parameters, Monte Carlo simulation study is performed.

## 1. Introduction

Burr type III distribution with two parameters was first introduced in the literature of Burr [1] for modelling lifetime data or survival data. It is more flexible and includes a variety of distributions with varying degrees of skewness and kurtosis. Burr type III distribution with two parameters *c* and *k*, which is denoted by BurrIII (*c*, *k*), has also been applied in areas of statistical modelling such as forestry (Gove et al. [2]), meteorology (Mielke [3]), and reliability (Mokhlis [4]).

The probability density function and the cumulative distribution function of BurrIII (*c*, *k*) are given by, respectively,

(1) $$\begin{array}{c} f\left(x\right)=ckx^{-(c+1)}{\left(1+x^{-c}\right)}^{-(k+1)},\;x>0,\;c>0,\;k>0,\\ F\left(x\right)={\left(1+x^{-c}\right)}^{-k}. \end{array}$$

Note that Burr type XII distribution can be derived from Burr type III distribution by replacing *X* with 1/*X*. The usefulness and properties of Burr distribution are discussed by Burr and Cislak [5] and Johnson et al. [6]. Abd-Elfattah and Alharbey [7] considered a Bayesian estimation for Burr type III distribution based on double censoring.

Order statistics are widely used in statistical modelling and inference. As a unified approach to a variety of models of ordered random variables such as ordinary order statistics, upper record values, and sequential order statistics, the concept of generalized order statistics (GOS) was introduced by Kamps [8]. Based on GOS, Burkschat et al. [9] introduced the concept of dual generalized order statistics as a dual model of GOS and a unification of several models of decreasingly ordered random variables such as reversed order statistics, lower record values, and lower Pfeifer records.

Let *F*(*x*) denote an absolutely continuous distribution function with the corresponding density function *f*(*x*) and let $X(r,n,\tilde{m},q)$, *r* = 1,2,…, *n*, be the corresponding dual GOS. Then, the joint probability density function of the first *n* dual GOS is the following:

(2) fX(1,n,m~,q),…,X(n,n,m~,q)(x1,…,xn)  =q(∏j=1n−1γj)[∏i=1n−1Fmi(xi)f(xi)]×Fq−1(xn)f(xn),

for *F* ^−1^(0) < *x*_*n*_ ≤ ⋯≤*x*_1_ < *F* ^−1^(1), $\tilde{m}=(m_{1},m_{2},\ldots ,m_{n-1})\in R^{n-1}$, with parameters *n* ∈ *N*, *n* ≥ 2, *q* > 0, and *M*_*r*_ = ∑_*j*=*r*_^*n*−1^ *m*_*j*_ such that *γ*_*r*_ = *q* + *n* − *r* + *M*_*r*_ > 0 for all *r* ∈ {1,2,…, *n*}. For simplicity, we shall assume *m*_1_ = *m*_2_ = ⋯ = *m*_*n*−1_ = *m*. If *m* = 0 and *q* = 1, then it gives the joint probability density function of *n* reversed order statistics from the independent and identically distributed (iid) random sample coming from *F*(*x*). If *m* = −1, then *X*(*r*, *n*, *m*, *q*) reduces to the *r*th lower *q*-record value of the iid random variables. Various distributional properties and some applications of the related topics are studied by Burkschat et al. [9], Ahsanullah [10], Jaheen [11], Mbah and Ahsanullah [12], Barakat and El-Adll [13], and W. Kim and C. Kim [14].

In this paper, our main objective is to describe MLE, the exact expression of the expected Fisher information matrix, and Bayes estimation procedures for the parameters of Burr type III distribution based on dual GOS, assuming the conjugate priors. In Section 2, we consider MLE and obtain an exact expression of the expected Fisher information matrix of the parameters. In Section 3, Lindley's approximation is used to obtain Bayes estimates for the parameters. Finally, in order to compare MLE with Bayes estimators, Monte Carlo simulation is studied in Section 4.

## 2. Maximum Likelihood Estimation

For *m*_1_ = *m*_2_ = ⋯ = *m*_*n*−1_ = *m*, suppose that *X*(1, *n*, *m*, *q*), *X*(2, *n*, *m*, *q*),…, and *X*(*n*, *n*, *m*, *q*) (*q* ≥ 1, *m* is a real number) are *n* dual generalized order statistics drawn from BurrIII (*c*, *k*). Using (1) and (2), we can get the following likelihood function:

(3) L(c,k ∣ x_)  =q(∏j=1n−1γj)[∏i=1nckxi−(c+1)(1+xi−c)−(k+1)]   ×[∏i=1n−1(1+xi−c)−mk](1+xn−c)−k(q−1)  =qcnkn(∏j=1n−1γj)[∏i=1n−1xick+cmk−1(1+xic)k+mk+1]xnckq−1(1+xnc)kq+1.

From (3), the log-likelihood function is proportional to

(4) l=ln⁡L(c,k ∣ x_)∝nln⁡c+nln⁡k +∑i=1n−1[(ck+cmk−1)ln⁡xi−(k+mk+1)ln⁡(1+xic)] +(ckq−1)ln⁡xn−(kq+1)ln⁡(1+xnc).

To derive the maximum likelihood estimators (MLE's) ${\hat{k}}_{M}$ and ${\hat{c}}_{M}$ of *k* and *c*,

(5) ∂l∂k=nk+(1+m)∑i=1n−1ln⁡ωi+qln⁡ωn,

(6) ∂l∂c=nc−∑i=1nxicυi+k[(1+m)∑i=1n−1υi+qυn],

where *ω*_*i*_ = *x*_*i*_^*c*^/(1 + *x*_*i*_^*c*^) and *υ*_*i*_ = ln⁡*x*_*i*_/(1 + *x*_*i*_^*c*^) for *i* = 1,2,…, *n*. From (5), MLE of *k* is expressed by

(7) $$\begin{array}{c} {\hat{k}}_{M}=-\frac{n}{\left(1+m\right)\sum_{i=1}^{n-1}\ln\omega_{i}+q\ln\omega_{n}}. \end{array}$$

Substituting (7) in (6), MLE ${\hat{c}}_{M}$ of *c* can be written as

(8) $$\begin{array}{c} \frac{n}{c}-\overset{n}{\underset{i=1}{\sum }}x_{i}^{c}\upsilon_{i}-\frac{n\left[\left(1+m\right)\sum_{i=1}^{n-1}\upsilon_{i}+q\upsilon_{n}\right]}{\left(1+m\right)\sum_{i=1}^{n-1}\ln\omega_{i}+q\ln\omega_{n}}=0. \end{array}$$

MLE of the parameter *c* is obtained by solving the nonlinear equation (8). Substituting MLE of the parameter *c* in (7), we can obtain MLE of the parameter *k*.

The asymptotic variance-covariance matrix of MLE for the parameters *k* and *c* is given by the elements of the Fisher information matrix:

(9) $$\begin{array}{c} I_{ij}=-E\left(\frac{\partial^{2}l\left(k,c\;|\;x\right)}{\partial k\partial c}\right),\;i,j=1,2. \end{array}$$

From (4), the asymptotic variance-covariance matrix for MLE is obtained by the following:

(10) Q∗=(Q11∗Q12∗Q12∗Q22∗)(k,c)=(k^,c^)−1=(−E(∂2l∂k2)−E(∂2l∂c∂k)−E(∂2l∂c∂k)−E(∂2l∂c2))(k,c)=(k^,c^)−1,

with

(11) ∂2l∂k2=−nk2,∂2l∂c∂k=(1+m)∑i=1n−1υi+qυn,∂2l∂c2=−nc2−(k+mk+1) ×∑i=1n−1υiωiln⁡xi−(kq+1)υnωnln⁡xn,

where *ω*_*i*_ = *x*_*i*_^*c*^/(1 + *x*_*i*_^*c*^) and *υ*_*i*_ = ln⁡*x*_*i*_/(1 + *x*_*i*_^*c*^).

Note that the Fisher information involves only a function of *X*_*i*_ and so we need the marginal probability density function of *i*th dual GOS based on the distribution function *F*(*x*) and the density function *f*(*x*). From Burkschat et al. [9], the marginal probability density function of *i*th dual GOS is the following:

(12) $$\begin{array}{c} f_{X(i,n,m,q)}\left(x_{i}\right)=\frac{C_{i-1}}{\Gamma \left(i\right)}{\left[F\left(x_{i}\right)\right]}^{\gamma_{i}-1}{\left[g_{m}\left(F\left(x_{i}\right)\right)\right]}^{i-1}f\left(x_{i}\right), \end{array}$$

where

(13) $$\begin{array}{c} C_{i-1}=\overset{i}{\underset{j=1}{\prod }}\gamma_{j},\;\;\;i=1,2,\ldots ,n,\\ h_{m}\left(x\right)=\{\begin{array}{cc} -\frac{1}{m+1}x^{m+1}, & m\neq -1\\ -\ln x, & m=-1, \end{array}\\ g_{m}\left(x\right)=h_{m}\left(x\right)-h_{m}\left(1\right),\;x\in \left[0,1\right). \end{array}$$

Assume that *m* ≠ −1. For each *i* = 1,2,…, *n*, we can have the expectation of *υ*_*i*_, which is

(14) E(υi)=E(ln⁡Xi1+Xic)=Ci−1(i−1)!∫0∞ln⁡xi1+xicF(xi)γi−1f(xi)gmi−1(F(xi))dxi.

If we use the transformation *z* = *F* ^1/*k*^(*x*_*i*_), then the expectation of *υ*_*i*_ is given by

(15) E(υi)=kCi−1c(i−1)!(m+1)i−1∑a=0i−1(i−1a)(−1)a ×{[k(γi+a(m+1))−1]×∑b=0∞(−1)b+1(1+b)2(k(γi+a(m+1))−1b)+1[k(γi+a(m+1))+1]2−1[k(γi+a(m+1))]2}.

To get the expectation of *υ*_*i*_ *ω*_*i*_ln⁡*X*_*i*_, we need to compute

(16) E(υiωiln⁡Xi) =E(Xic(ln⁡Xi)2(1+Xic)2) =Ci−1(i−1)!∫0∞xic(ln⁡xi)2(1+xic)2F(xi)γi−1f(xi)gmi−1(F(xi))dxi.

By the same transformation method *z* = *F* ^1/*k*^(*x*_*i*_), the expectation of *υ*_*i*_ *ω*_*i*_ln⁡*X*_*i*_, for *m* ≠ −1, is given by

(17) E(υiωiln⁡Xi)=2kCi−1c2(i−1)!(m+1)i−1∑a=0i−1(i−1a)(−1)a ×{1[k(γi+a(m+1))+1]3−1[k(γi+a(m+1))+2]3+∑n=1∞[1n[k(γi+a(m+1))+n+2]2−1n[k(γi+a(m+1))+n+1]2]−k(γi+a(m+1))×∑b=0∞(−1)b(1+b)3(k(γi+a(m+1))b)}.

For *m* = −1, we can compute the expectation of *υ*_*i*_, which is

(18) E(υi)=E(ln⁡Xi1+Xic)=Ci−1(i−1)!∫0∞ln⁡xi1+xicF(xi)γi−1f(xi)g−1i−1(F(xi))dxi.

Using the same transformation method *z* = *F* ^1/*k*^(*x*_*i*_), the expectation of *υ*_*i*_ is the following:

(19) E(υi)=i(kq)ic(kq+1)i+1−ickq +(kq)ic∑n=1∞{1n(n+kq)i−1n(n+kq+1)i}.

To get the expectation of *υ*_*i*_ *ω*_*i*_ln⁡*X*_*i*_, we should compute

(20) E(υiωiln⁡Xi) =E(Xic(ln⁡Xi)2(1+Xic)2) =Ci−1(i−1)!∫0∞xic(ln⁡xi)2(1+xic)2F(xi)γi−1f(xi)g−1i−1(F(xi))dxi.

With the transformation *z* = *F* ^1/*k*^(*x*_*i*_) and (∑_*n*=1_^*∞*^(*z* ^*n*^/*n*))^2^ = ∑_*n*=2_^*∞*^∑_*j*=1_^*n*−1^(*z* ^*n*^/(*n* − *j*)*j*), the expectation of *υ*_*i*_ *ω*_*i*_ln⁡*X*_*i*_, when *m* = −1, is given by

(21) E(υiωiln⁡Xi)=(kq)ic2{i(i+1)(kq+1)i+2−i(i+1)(kq+2)i+2+∑n=2∞∑j=1n−1(1j(n−j)(n+kq+1)i−1j(n−j)(n+kq+2)i)+2i∑n=1∞(1n(n+kq+2)i+1−1n(n+kq+1)i+1)}.

Now, we can get each entry of the Fisher information matrix *Q* ^*^ as follows:

(22) Q11∗=−E(∂2l∂k2)=nk2,Q12∗=Q21∗=−E(∂2l∂c∂k)=−(1+m)∑i=1n−1E(υi)−qE(υn),Q22∗=−E(∂2l∂c2)=nc2+(k+mk+1)×∑i=1n−1E(υiωiln⁡Xi)+(kq+1)E(υnωnln⁡Xn).

Using (15), (17), (19), and (21), all entries *Q*_11_^*^, *Q*_12_^*^, *Q*_22_^*^ can be explicitly expressed, depending on *m*.

## 3. Bayes Estimation 

In this section, we want to estimate the parameters *c* and *k* under squared error loss (SEL) function, which is defined as $L_{0}(\theta ,\hat{\theta })={\left(\theta -\hat{\theta }\right)}^{2}$ for a parameter *θ*. Assuming that the parameters *c* and *k* are unknown, a natural choice for the prior distributions of *k* and *c* would be to assume that the two quantities are independent gamma distributions as in the following:

(23) $$\begin{array}{c} \pi \left(c,k\right)=\pi_{1}\left(k\right)\pi_{2}\left(c\right), \end{array}$$

where

(24) $$\begin{array}{c} \pi_{1}\left(k\right)=\frac{\beta^{-\alpha }}{\Gamma \left(\alpha \right)}k^{\alpha -1}e^{-k/\beta },\\ \pi_{2}\left(c\right)=\frac{\delta^{-\gamma }}{\Gamma \left(\gamma \right)}c^{\gamma -1}e^{-c/\delta }, \end{array}$$

where *α*, *β*, *γ*, and *δ* are chosen to reflect prior knowledge about *k* and *c*.

By combining (3) and (24), the joint posterior density function of *c* and *k* can be put as follows:

(25) π(c,k ∣ x_)∝cn+γ−1kn+α−1e−k/β−c/δ ×(∏i=1n−1xick+cmk−1(1+xic)k+mk+1)xnckq−1(1+xnc)kq+1.

Under the SEL function, it is well known that the Bayes estimator of a function *U* = *U*(*k*, *c*) is the posterior mean of the function, which is

(26) U^B=E[U(k,c) ∣ x_]=∬0∞U(k,c)L(c,k ∣ x_)π(c,k)dc dk∬0∞L(c,k ∣ x_)π(c,k)dc dk.

In general, the integral ratio in (26) cannot be expressed in a simple closed form. Hence, we use Lindley's approximation [15] to obtain a numerical approximation. In a two-parameter case, say (*λ*_1_, *λ*_2_) = (*k*, *c*), based on Lindley's approximation, the approximate Bayes estimator of a function *U* = *U*(*λ*_1_, *λ*_2_), under the SEL function, leads to

(27) U^B=U(λ1,λ2)+12(A+l30∗B12+l03∗B21+l21∗C12+l12∗C21) +p1A12+p2A21,

where

(28) A=∑i=12∑j=12Uijτij,  lij∗=∂i+jl∂λ1i∂λ2j;  i,j=0,1,2,3, with  i+j=3,pi=∂p∂λi,  Ui=∂U∂λi,  Uij=∂2U∂λi∂λj,p=ln⁡π(λ1,λ2), for  i,j=1,2,

and for *i* ≠ *j*,

(29) $$\begin{array}{c} A_{ij}=U_{i}\tau_{ii}+U_{j}\tau_{ji},\;\;B_{ij}=\left(U_{i}\tau_{ii}+U_{j}\tau_{ij}\right)\tau_{ii},\\ C_{ij}=3U_{i}\tau_{ii}\tau_{ij}+U_{j}\left(\tau_{ii}\tau_{jj}+2\tau_{ij}^{2}\right). \end{array}$$

Note that *τ*_*ij*_ is the (*i*, *j*)th element of the inverse of the matrix (*l*_*ij*_), *i*, *j* = 1,2, where *l*_*ij*_ = ∂^2^ *l*/∂*λ*_1_∂*λ*_2_. Moreover, (27) is to be evaluated at the MLE's of *λ*_1_ and *λ*_2_.

Now, we apply Lindley's approximation (27) to our case, where (*λ*_1_, *λ*_2_) = (*k*, *c*) and *U*(*λ*_1_, *λ*_2_) = *U*(*k*, *c*). The elements *τ*_*ij*_ can be obtained as

(30) (−l11−l12−l21−l22)−1=1l11l22−(l12)2(−l22l12l21−l11)=(τ11τ12τ21τ22).

Let *H* = −*l*_22_, *G* = −*l*_11_, and *I* = *l*_12_ = *l*_21_. Then, *N* = *GH* − *I* ^2^. Then, we can rewrite this as

(31) $$\begin{array}{c} \tau_{11}=\frac{H}{N},\;\;\tau_{12}=\tau_{21}=\frac{I}{N},\;\;\tau_{22}=\frac{G}{N}, \end{array}$$

where

(32) G=nk2,H=nc2+(k+mk+1) ×∑i=1n−1υiωiln⁡xi+(kq+1)υnωnln⁡xn,I=(1+m)∑i=1n−1υi+qυn.

Also, the values of *l*_ij_^*^ can be obtained as follows for *i*, *j* = 0, 1, 2, 3;

(33) l30∗=∂3l∂k3=2nk3,l03∗=∂3l∂c3=2nc3−∑i=1nωiυi2(1−xic)ln⁡xi −k(1+m)∑i=1n−1ωiυi2(1−xic)ln⁡xi −kqωnυn2(1−xnc)ln⁡xn,l21∗=∂3l∂k2∂c=0,l12∗=∂3l∂k∂c2=−(m+1)∑i=1n−1ωiυiln⁡xi−qωnυnln⁡xn.

Note *p* = ln⁡*π*(*k*, *c*)∝(*α* − 1)ln⁡*k* + (*γ* − 1)ln⁡*c* − *k*/*β* − *c*/*δ*. Then, we get

(34) $$\begin{array}{c} p_{1}=\frac{\partial p}{\partial k}=\frac{\alpha -1}{k}-\frac{1}{\beta },\;\;p_{2}=\frac{\partial p}{\partial k}=\frac{\gamma -1}{c}-\frac{1}{\delta }. \end{array}$$

Substituting all the above components to (27), the Bayes estimate of the function *U*(*k*, *c*) given in (27), under the SEL function, becomes

(35) $$\begin{array}{c} {\hat{U}}_{B}=E\left[U\left(k,c\right)\;|\;\underline{x}\right]=U+\psi_{0}+\psi_{1}U_{1}+\psi_{2}U_{2}, \end{array}$$

where

(36) ψ0=12N(U11H+U12I+U21I+U22G),ψ1=l30∗H22N2+l03∗GI2N2+l12∗(HG+2I2)2N2+p1HN+p2IN,ψ2=l30∗HI2N2+l03∗G22N2+3l12∗GI2N2+p1IN+p2GN.

From (35), we can deduce the values of the Bayes estimates of the parameters *c* and *k* as follows.

If *U*(*k*, *c*) = *k*, then *ψ*_0_ = 0, *U*_1_ = 1, and *U*_2_ = 0. Hence,

(37) $$\begin{array}{c} {\hat{k}}_{B}=k+\psi_{1}. \end{array}$$

If *U*(*k*, *c*) = *c*, then *ψ*_0_ = 0, *U*_1_ = 0, and *U*_2_ = 1. Hence,

(38) $$\begin{array}{c} {\hat{c}}_{B}=c+\psi_{2}. \end{array}$$

Note that (35), (37), and (38) are to be evaluated at MLE's $\left({\hat{k}}_{M},{\hat{c}}_{M}\right)$.

## 4. Simulation Study and Comparisons 

In this section, we consider MLE and the approximate Bayes estimates for two parameters *c* and *k* of Burr type III distribution. To assess the performance of these estimates, we conducted a simulation study.

Let *X*_*L*(1)_ = *x*_1_, *X*_*L*(2)_ = *x*_2_,…, and *X*_*L*(*n*)_ = *x*_*n*_ be the lower record values of size *n* which can be obtained from the dual GOS scheme as a special case by taking *m* = −1 and *q* = 1. MLE and Bayes estimates for the parameters of BurrIII (*c*, *k*) based on lower records are computed and compared through the Monte Carlo simulation study according to the following steps.

(1) For *k* = 2 and *c* = 3, samples of lower record values of size *n* (*n* = 4,6, 8,10) were generated from Burr type III distribution. Burr type III lower record values are generated using the inverse cdf, *X*_*i*_ = (*u*_*i*_^−1/*k*^ − 1)^−1/*c*^, where *u*_*i*_ is the uniformly distributed random variate.
(2) MLE's, ${\hat{k}}_{M}$ and ${\hat{c}}_{M}$ of the parameters *k* and *c*, are calculated by iteratively solving (7) and (8) with *m* = −1 and *q* = 1.
(3) For given vlaues of prior parameters (*α*, *β*, *γ*, *δ*), the Bayes estimates of *k* and *c* are computed from (37) and (38) with *m* = −1 and *q* = 1.
(4) The above steps are repeated 1,000 times to evaluate the root mean squared error (RMSE) of MLE and Bayes estimates for the different sample sizes *n*. Note that
  (39) $$\begin{array}{c} \text{RMSE}=\sqrt{\frac{1}{1000}\overset{1000}{\underset{i=1}{\sum }}{\left(g\left(\theta_{0}\right)-g\left({\hat{\theta }}_{i}\right)\right)}^{2}}, \end{array}$$
  where *g*(*θ*_0_) is the true value and $g({\hat{\theta }}_{i})$ is the *i*th estimate of *g*(*θ*) evaluated at $\hat{\theta }$.

Table 1 provides the averaged RMSE of MLE and Bayes estimates based on lower record values for two sets of prior parameters (*α*, *β*, *γ*, *δ*). To show the consistency of the result across varying data sets with large variability and differing sample sizes, we simulate data under two sets of parameters, each prior distribution with large variability. We see that the Bayes estimates are better than MLE in the sense of comparing RMSE of the estimates. As the sample size *n* increases, RMSE of the estimates should decrease, which is the case in our computer simulation.

## Figures and Tables

**Table 1:** The averaged RMSE for MLE and Bayes Estimates of the parameters k and c for different n.

*n*	RMSE					
	${\hat{c}}_{M}$	${\hat{k}}_{M}$	${\hat{c}}_{B}$	${\hat{k}}_{B}$	${\hat{c}}_{B}$	${\hat{k}}_{B}$
			(*α* = 3, *β* = 2	*γ* = 2, *δ* = 3)	(*α* = 5, *β* = 1	*γ* = 2, *δ* = 5)
4	2.0268	2.6734	1.7365	2.3706	1.9826	1.7875
6	1.6421	2.1182	1.3672	1.9863	1.5851	1.4074
8	1.4473	1.8153	1.1980	1.7572	1.3504	1.3357
10	1.3556	1.5863	1.1267	1.5180	1.3264	1.2318
